# Ethics 4.0: Ethical Dilemmas in Healthcare Mediated by Social Robots

**DOI:** 10.1007/s12369-023-00983-5

**Published:** 2023-03-07

**Authors:** Antonio Soares, Nuno Piçarra, Jean-Christophe Giger, Raquel Oliveira, Patrícia Arriaga

**Affiliations:** 1 ISCTE-Instituto Universitário de Lisboa, CIS-IUL, Lisboa, Portugal; 2 Psychology Research Centre (CIP), University of Algarve, Faro, Portugal

**Keywords:** Moral judgments, Nursing robots, Warmth, Competence, Trustworthiness, Health framing

## Abstract

This study examined people’s moral judgments and trait perception toward a healthcare agent’s response to a patient who refuses to take medication. A sample of 524 participants was randomly assigned to one of eight vignettes in which the type of healthcare agent (human vs. robot), the use of a health message framing (emphasizing health-losses for not taking vs. health-gains in taking the medication), and the ethical decision (respect the autonomy vs. beneficence/nonmaleficence) were manipulated to investigate their effects on moral judgments (acceptance and responsibility) and traits perception (warmth, competence, trustworthiness). The results indicated that moral acceptance was higher when the agents respected the patient’s autonomy than when the agents prioritized beneficence/nonmaleficence. Moral responsibility and perceived warmth were higher for the human agent than for the robot, and the agent who respected the patient’s autonomy was perceived as warmer, but less competent and trustworthy than the agent who decided for the patient’s beneficence/nonmaleficence. Agents who prioritized beneficence/nonmaleficence and framed the health gains were also perceived as more trustworthy. Our findings contribute to the understanding of moral judgments in the healthcare domain mediated by both healthcare humans and artificial agents.

## Introduction

Imagine that you are responsible for the healthcare of a patient who refuses to take medication. Would you accept or decline the patient’s decision? Would you try to persuade the patient to take medication by framing your argument about the health gains of taking the medication or the health losses of refusing the medication? What if the health caregiver was a socially assistive robot? To explore these questions, we developed a set of vignettes with different solutions to this dilemma and asked participants to judge a healthcare agent (human or robot) regarding the moral acceptance of her/its behavior, attributions of moral responsibility, warmth, competence [1, 2], and trustworthiness [3, 4] attributes, which are considered central for healthcare interactions [5, 6]. In these vignettes, we also analyzed the effects of using different health persuasive framings (gain or loss) in combination with an ethical decision, in which the healthcare agent has accepted the patient decision to refuse medication (respecting the patient autonomy) or has not accepted (by prioritizing the beneficence and nonmaleficence of the patient health). These principles have also been considered the most relevant in bioethics [7]. The autonomy principle refers to respecting the patient’s right to freely control his/her own health outcomes, whereas the beneficence principle involves the healthcare actions intended to maximize the patient’s health, and nonmaleficence refers to the duty to not cause harm nor impose risks of harm to patients [7, 8]. In our study, we combined the beneficence/nonmaleficence principles, since not accepting the patient’s refusal to take medication is expected to lead to health benefits and the avoidance of harm [7].

Our study extends previous research [9], focusing on a potential healthcare situation in which a patient refuses to take medication. We tested how different health framing messages to persuade patients to take medication in combination with distinct ethical decisions impact people’s moral judgments and perceptions of the healthcare agent traits (warmth, competence, trustworthiness).

### Artificial Intelligence & Social Robots in Healthcare

The field of Artificial Intelligence (AI) is concerned with understanding and building intelligent machines, capable of estimating how to act effectively and safely in a wide variety of situations [10], including healthcare settings, in which AI is expanding into areas such as treatment selection, patient monitoring, and targeting drug prioritization at the support level. There is the prospect that automation in medicine may increase productivity by streamlining human routines and decisions [11], and that robots will be used in this field and will soon be able to operate fully autonomously [12]. Robots are embodied agents that can be equipped with processors, sensors, and effectors (e.g., legs, wheels, claws) to be able to operate in the environment, manipulate the physical world and perform tasks [10]. In addition, social robots can be equipped with AI to interact socially with humans through features such as recognition of emotional prototypical configurations, social learning, imitation, verbal, and nonverbal communication [13]. Some authors have suggested that routine healthcare requiring only standardized procedures could be better performed by a robot [14], while others have also pointed out that nurses might be replaced by social humanoid robots with AI, taking over prediction and prescription in healthcare [15]. There is also evidence that the physical presence of a social robot can have a social facilitation effect [16].

The systematic review by Esterwood and Robert [17] suggests that studies investigating how robots in healthcare settings are perceived tend to focus on performance, acceptance, and social/emotional outcomes, while perceptions of a robot’s traits or anthropomorphism have been less examined. Thus, focusing on how humans perceive robotic traits by also considering the context and the tasks they were programmed to perform are relevant because this may bring insights for design recommendations. Some authors recognize that performing nursing activities requires specific human attributes, such as feelings of concern, sympathy, and empathy [15], but other authors sustain that technology already simulates such attributes, by building robots with theory of mind that enable them to express feelings [for a review [18]]. In this sense, it is possible that embodied robots may represent a new ontological category [19], based on the idea that people tend to ascribe attributes such as mental states, sociability, and even moral traits to embodied robots. Thus, will people attribute warmth, competence, and trustworthiness to robots in healthcare as they do to human beings?

### Agent-Patient Relationship in Healthcare, Bioethics and Adherence

Interventions to encourage patient adherence to medical recommendations are the most likely path to positive patient outcomes. However, there is a great variability in medical diagnosis and therapeutic interventions which may lead patients eventually to non-adherence to prescribed medications. Non-adherence to medication is often related to patients not following the complete prescribed regiment or not taking the medication in the proscribed dosage, with the consequence of not receiving full benefits [20], such as the cases of chronic patients, who are often the beneficiaries of home healthcare [21].

Indeed, patients seem to be taking more active roles [20, 22] in their treatments, which also raises new concerns about some of the ethical dilemmas in healthcare practice, such as the case between autonomy and beneficence/nonmaleficence [8]. The conflict with the principles of beneficence/nonmaleficence arises when the duty of respect for autonomy may result in harm, or lack of benefit, to the health of the person in reference.

The communication between healthcare agents and patients and the role each plays in decision-making may impact the patient’s health outcomes. Between healthcare agent and patient, there are three relationship types: paternalism, mutuality, and consumerism. Paternalism is the paradigm in which the healthcare agent makes most decisions, whereas in mutuality the healthcare agent and the patient have a balanced status in decision-making. In contrast, in consumerism the decision-making is the sole responsibility of the patient [20]. These relationship models (paternalism, mutuality, and consumerism) may have implications for the judgement of moral acceptance and attribution of responsibility, especially regarding the authority status vis-à-vis the ethical principles of respect for autonomy and beneficence/nonmaleficence. Thus, a central problem in bioethics is whether the respect for the patient autonomy should take priority over beneficence and/or nonmaleficence for those patients. To solve some ethical dilemmas, healthcare professionals must follow to the ethical code of conduct of their profession and take an Oath stating that they will follow their professional ethical standards. Doctors take the Hippocratic Oath, and nursing schools in many countries, including Portugal and Brazil, take the Florence Nightingale Pledge [23, 24], whose values include the interests of patients over medical orders [25]. The International Council of Nurses (ICN) has also developed the ICN Code of Ethics for Nurses which defines and guides nurse’s ethical decision-making practices worldwide [26]. In particular, according to the autonomy principle, patients have the right to make decisions about their medical care, including the refusal of any type of medical treatment; thus, nurses should respect their choices and not attempt to go against their will. However, nurses should also consider if the patient has cognitive or affective impairments that might compromise his/her capacity to make decisions, suggesting that self-determination may not be absolute in all cases. In addition, nurses should provide the information needed to allow any patient to make the best decision [26]. According to the guidelines of the National Institute for Health and Clinical Excellence on medication adherence, non-adherence should be recognized as a very common phenomenon [27]. Moreover, information about the risks and benefits of the patient choices should be provided by the healthcare, and shared-decision making is very valued by patients [27]. Therefore, effective communication between healthcare professionals and patients is considered very important to address these situations.

One approach to communication in healthcare is the message framing perspective, proposed by Rothman and Salovey [28]. Gain framed messages emphasize the advantages of compliance, whereas loss-frame messages highlight the disadvantages of non-compliance. Message framing draws from the framework provided by prospect theory [29], suggesting that in low-risk situations potential gains can be more motivating than potential losses, whereas in situations involving high-risk, potential losses may be more motivating. Translating this to health, behaviors about enhancing or maintaining health (preventive behaviors) should be easier to promote with messages highlighting potential gains and benefits. On the other hand, behaviors that lead to stopping or reducing health problems (detection behaviors) should be easier to promote with messages emphasizing potential losses. However, the findings of meta-analytic reviews showed a more complex picture. Although meta-analytic studies found that gain messages were better at promoting prevention behaviors [30], and in some studies loss messages better promoted detection behaviors [31], the results also seem to depend on specific health-domains [30], the outcome measure (e.g., behavioral intention, actual behavior) [32], and, in general, the effect sizes have been small. Empirical studies have also shown that individuals’ choices, when faced with messages in a setting involving some gain, are generally oriented toward the safer alternative, whereas a setting involving some loss tends to evoke a riskier option. Furthermore, a given loss may be weighted by individuals as more significant than an equivalent gain, that is, people’s response and displeasure associated with potential losses may be greater than the pleasure and reaction in the face of the likelihood of similar gains, a principle known as risk aversion [29]. Thus, it will be important to investigate the perceived judgments of a healthcare agent when using a persuasive gain versus a loss framed argument to a patient who explicitly does not want to adhere to prescribed medication.

The ethical virtue theory sustains that the agent’s attributes are important to identify and perform morally correct actions [8]. The importance of how individuals perceive certain attributes of another individual has also been highlighted by the stereotype content model [1, 2], which emphasize two dimensions: warmth and competence. Indeed, among the traits more frequently studied, the attributes associated with warmth (e.g., sensibility, patience) and competence have been considered the most relevant. Dai and MacDorman [33], for example, have shown that the higher the perception of warmth and competence in a healthcare agent, the higher the individual’s intention to adhere to the healthcare. Also important is to be perceived as trustworthiness since it is essential to establish of a relation [6].

### Dilemmas with Robotic Agents

To the best of our knowledge, sacrificial dilemmas in scenarios involving robots have been the most frequently studied. Malle et al. [34], for example, compared people’s moral judgments of permissibility, wrongness, and blame about the actions of a robotic agents in comparison to a human agent in a moral dilemma involving acting and sacrificing one person to save four people, or do nothing and let them get hit by a trolley. Participants considered robots to be more expected to sacrifice one person for the good of others (“utilitarian” choice) when compared to human agents, although utilitarian sacrifice was considered generally permissible for human agents. However, human agents were assigned greater blame when they decided to sacrifice than when they chose to do nothing. In contrast, greater blame was attributed to robots than to human agents under the condition in which the agent decided to do nothing. In another study, also using the trolley dilemma and different pictorial robotic prototypes (mechanical, humanoid, and AI), Malle et al. [35] found that mechanical robots that made an utilitarian decision were less blamed than those who choose inaction. In contrast, humans were blamed more for action than for inaction. Moreover, agents who decided to act in a utilitarian way were rated more positively than those who did not act, and human agents were rated more positively than any of the artificial agents, regardless of the agents’ decisions.

The increasing use of assistive robots in healthcare requires studies to gauge people’s judgments regarding situations in which their ethical decisions may impact patients’ well-being. However, there is scant moral psychological research about nursing robotics. Anderson and colleagues [36, 37] has been one of the few authors discussing dilemmas applied to healthcare robots, mainly involving the ethical principles of beneficence, nonmaleficence, and respect for the patient autonomy. These dilemmas typically involve a healthcare agent recommending medication to a patient, although the human patient rejects the treatment. Similarly, Sasson [38] also discussed ethical decisions involving an institutionalized patient with preserved mental faculties that require healthcare assistance but also resisting the treatment. However, to the best of our knowledge only Laakasuo et al. [9] have empirically studied how people judge these ethical dilemmas in healthcare. In six studies (five experiments and one field study), participants were exposed to hypothetical scenarios in which an agent (human or robot) is instructed to forcefully medicate a patient who refuses medication, and then decides to either respect the patient autonomy or to forcefully medicate the patient. They measured participants’ judgments of moral acceptance, the attributions of responsibility and the trustworthiness of the healthcare agent, in relation to the ethical decision. It was found that moral acceptance attributed to respecting autonomy did not vary as a function of the type of agent, although robots were judged to be significantly less morally acceptable than humans when forcefully medicating the patient. Their results also showed significantly higher attribution of moral responsibility to the human relative to the robotic agent, regardless of the ethical decision. In addition, they found that human agents were rated as more trustworthy than robots and no differences were found for trustworthiness as a function of the ethical decision. However, other questions remain unanswered: Since the patient’s autonomy should be respected by healthcare professionals, even in cases of non-adherence to prescribed medication, how should the healthcare agent communicate the need to take the medication to a patient? Should the healthcare agent emphasize either the gains of taking the medication or the losses of non-adherence? Finally, how do people imagine the physical appearance of a robotic healthcare agent who needs to make an ethical decision?

## Aims and Hypothesis

Our study addresses how people judge a healthcare agent’s reaction to a patient who refuses to take medication, as a function of the healthcare agent (human vs. robot), the health framing argument (gain vs. loss), and the ethical decision (respect for autonomy vs. beneficence/nonmaleficence). We adapted the scenario used in Laakasuo et al. [9] study, which was also discussed previously by Anderson et al. [36, 37], in which the ethical principles of beneficence, nonmaleficence, and respect for patient autonomy were considered, involving a patient who does not adhere to medication. However, some modifications were made, including the way ethical decisions were made (i.e., we chose not to force the patient to medicate), and by adding a gain-loss framing message explaining to the patient about the consequences of not accepting the medication, which to our knowledge, had not yet been investigated in these dilemmas. We examined whether these independent variables influence participants’ judgments about the moral acceptability of the decision made, the moral responsibility of the healthcare agent, and the perception of her attributes of warmth, competence, and trustworthiness.

Because several individual variables, such as negative attitudes towards robots [39], moral concerns [40], and engagement with one’s own health [41], might be correlated with the dependent variables, we use them as covariates. In addition, since the data were collected in two different countries, Portugal and Brazil, we will consider the country as a potential covariate if the results suggest country differences on some of the outcomes. However, we do not expect cultural differences, given that these two countries have historical roots, share the same language and other cultural affinities (e.g., historically catholic, traditional values) [42]. Both countries also seem to report relatively similar acceptability judgments towards sacrificial dilemmas such as the trolley problem [43]. In addition, based on the dataset shared by the Wellcome Global Monitor [44], which addresses how people think about major science and health challenges worldwide, most participants from both countries also expressed high levels of trust in doctors and nurses, and in their medical and health advice, although the percentages were slightly higher for the Portuguese respondents (88%) than for the Brazilians (71–72%). In addition, a recent study [45] indicates that people’s first impressions about the attributes of others, such as trustworthiness, competence, and caring, seem to be more affected by the perceiver’s own individual characteristics than by their culture background.

In addition, we will explore the degree of human likeness attributed to the robotic healthcare agent and examine which robot would be considered suitable to perform the professional health work. We will also analyze how the degree of human likeness of the imagined robotic nurse relates with perceived attributes and moral judgments, given that previous research suggests that robot appearance affects moral judgments [46] and user’s perceptions of the robot’s qualities [47, 48].

Since our line of work is relatively recent, we base our hypotheses on the recent findings of Laakasuo et al. [9], by anticipating the following:

H1. Participants in the human agent condition will attribute higher levels of moral acceptability (H1a), moral responsibility (H1b), warmth (H1c), competence (H1d), and trustworthiness (H1e) than participants in the robot agent condition.

H2. Participants in the robot agent condition will attribute higher levels of moral acceptability to the robot that respects the patient’s autonomy than to the robot that does not accept this autonomy.

The remaining analyses, including the effects of the gain-loss framing on the outcomes will be exploratory given the inconsistency of prior results in healthcare, and the absence of prior experimental manipulation in scenarios using robots.

We have preregistered the main aim and hypothesis of the current study at AsPredicted.org (https://aspredicted.org/cq8gw.pdf).

## Method

### Participants

Sample size was estimated using GPower3.1.9.4 [49]. The estimated minimum sample size was 469 participants, considering medium to low effect sizes (*f* = 0.15), power of 90%, and alpha 0.05 for the analysis of covariance (ANCOVAs) with three between-subjects variables.

Participants were recruited through convenience and snowballing procedures, via social networks and among acquaintances. A total of 841 participants voluntarily participated in the study, of which 317 participants were removed from the analysis because they missed more than 50% of the responses (*n* = 223, 26.50%), and failed the attention check question (*n* = 94, 11.20%). The final sample was composed of 524 participants of whom 316 were Brazilians and were 179 Portuguese. The participants’ age ranged from 18 to 77 years (*M* = 38.73, *SD* = 14.22), with a predominance of women (*n* = 350, 70%). Most participants were either single (45.70%) or married/in a stable union (42.10%), mostly with a bachelor’s degree (*n* = 348, 69.46%). Several participants reported being students as their main occupation (23.44%), followed by professionals in the fields of health (19.09%), education (10.58%), administration (9.13%), and law (5.39%). The majority reported their general health as good (60.1%) or very good (19.8%). Many participants (43.9%) stated that they had already provided home healthcare assistance to someone, while only 11.5% stated that they had already needed healthcare assistance for themselves (see Table 1).

**Table 1:** Sociodemographic Characteristics of the Participants

		*n*	%			*n*	%
Sex				Educational level			
	Female	350	66.8		Middleschool	5	1
	Male	150	28.6		Highschool	61	11.6
	Not reported	24	4.6		Bachelor / University degree	206	39.3
Nationality					Post-graduate degree	142	27.1
	Portuguese	179	34.2		Masters’ degree	67	12.8
	Brazilian	316	60.3		Doctoral degree	20	3.8
	Not reported	29	5.5		Not reported	23	4.4
Marital status				Health			
	Married / Stable relationship	211	40.3		Very good	99	18.8
	Widower	8	1.5		Good	300	57.3
	Divorced / Separated	46	8.8		Resonable	94	17.9
	Single	229	43.7		Bad	5	1
	Other	7	1.3		Very bad	1	0.2
	Not reported	23	4.4		Not reported	25	4.8

*Note*. *N* = 524. Participants were on average 38.7 years old (*SD* = 14.2). Age varied between 18 and 77 years.

### Measures

#### Independent Variables

Participants were randomly assigned to one of eight different vignettes, with each vignette being shown to between 60 and 72 participants. We adapted the Laakasuo et al. [9] vignettes by manipulating the healthcare agent (human vs. robot) and the ethical decision (respect for autonomy vs. beneficence/nonmaleficence) in a situation where a patient refuses medication. In addition, we manipulated the health framing (gain vs. loss). Thus, three variables were manipulated with a 2 (healthcare agent) X 2 (health framing) X 2 (ethical decision) between-subjects factorial design. These variables were dummy coded as follows: healthcare agent (-1 = human; 1 = robot), ethical decision (-1 = respect for autonomy; 1 = beneficence/nonmaleficence), and health framing (-1 = gain; 1 = loss). Each survey included one of these eight combinations of the manipulated variables. The healthcare agent was female and named “Lena” in all conditions. The patient was also a woman (Mrs. M.). The manipulations are in square brackets in the following vignette, but can also be found in detail at the Open Science Framework project page (https://doi.org/10.17605/OSF.IO/2F9QJ):

“We are in the year 2035. Lena is a [nurse | nursing robot] who follows medical instructions, programmed through an Artificial Intelligence (Health-AI) system. Lena has been hired to provide homecare for a patient who has a physical health problem (without any intellectual/cognitive problems). One day the patient refuses to take a medication because it is unpleasant. The [nurse | nursing robot] Lena emphasizes the [gains | losses] of [taking | not taking] the prescribed medication, saying: - It is [beneficial | harmful] for your health [to take | not to take] all the medication because it [benefits | harms] your immunity and physical stamina. [However, I respect your decision not to take the medication | Therefore, I do not accept your decision, you will have to take the medication].“

#### Dependent Variables

**Moral acceptance** of the healthcare agent behavior was measured with 16 items, based on the items used by Laakasuo et al. [9]. The item “the nurse | nursing robot is a relied upon member of the medical staff” was not included because our vignettes did not mention a medical team. In addition, we split the item “the nurse | nursing robot is sympathetic or nice” into two, as follows: “Lena is sympathetic” and “Lena is nice”, because they might be interpreted differently in our vignettes. The 16 items were answered on a 7-point scale (1 = Completely disagree, 7 = Completely agree). In our study an α = 0.90 was obtained. Thus, a moral acceptance composite score was calculated by averaging 16 items. Higher scores indicate strong acceptance of the agent’s behavior.

Attribution of **moral responsibility** to the healthcare agent was measured with three items (e.g., “Lena is responsible for the decision it/she made.“) based on Laakasuo et al. [9]. These items were answered on a 7-point scale (1 = Completely disagree, 7 = Completely agree) and achieved a good internal reliability (α = 0.81). A composite of moral responsibility was calculated by averaging the three items, with higher scores indicate more attribution of moral responsibility to the healthcare agent.

To measure the perceived **warmth and competence** attributes of the healthcare agent we relied on the Stereotype Content Model [1, 2], and adapted the items already used in previous studies conducted in Portuguese samples [50, 51]. Four items were initially used to measure warmth: warm, kind, friendly, and well-intentioned, however, the item “well-intentioned” was excluded from analysis since it showed an inter-item correlation < 0.40 [52]. The 3-item of the warmth measure showed good internal reliability (α = 0.89). Competence was measured with six items: competent, capable, intelligent, efficient, skillful, and confident, obtaining an α = 0.90. The measure of **trustworthiness** was adapted from [3, 4] studies and consisted of three items: reliable, sincere, and honest, achieving an α = 0.78. All the responses were provided on the same 7-point scale (1 = Completely disagree, 7 = Completely agree). The average for each attribute was used to calculate each measure, with higher scores indicating higher perceptions of the agent’s warmth, competence, and trustworthiness.

#### Additional Measures

We used the Portuguese 12-item version of the **Negative Attitudes towards Robots** (NAR) Scale[53]. The responses to each item (e.g., “I would feel relaxed talking with robots”) were made on a 7-point scale (1 = Completely disagree, 7 = Completely agree) and achieved an α = 0.88. A composite was calculated by averaging the 12-items, with higher scores indicating higher negative attitudes towards robots.

**Moral concerns** of harming or treating others unfairly are the most widespread and often included in measures of morality. Thus, we measured these two individual-focused concerns of the moral relevance domain from the Moral Foundations Questionnaire [54]: harm (e.g., “Whether or not someone suffered emotionally”) and fairness (e.g., “Whether or not someone acted unfairly”), each dimension with three items. Both dimensions were measured using a 6-point scale (1 = not at all relevant, 6 = extremely relevant) and achieved acceptable internal reliability scores (α = 0.79 for harm and 0.78 for fairness). The items for each measure were averaged, with higher scores indicating higher relevance attributed to harm or fairness concerns.

To measure participant’s **engagement in own’s health**, we relied on the following three dimensions of the Altarum Consumer Engagement Measure [55]: “Commitment”, corresponding to proactive self-care behaviors by adhering to a healthy lifestyle (6 items, e.g. “I take an active role in my own health care”); “Navigation”, corresponding to the ability to participate in decisions related to own’s health treatments (5 items, e.g. “I have lots of experience using the healthcare system”), and ownership, corresponding to the responsibility for one’s health (5 items, e.g. “My health is my responsibility, not someone else’s”), which were combined into a single composite of health engagement with 16 items, obtaining an α = 0.83. Each item was responded to on a 5-point scale (1 = strongly disagree, 5 = strongly agree). The items were averaged, with higher scores corresponding to higher levels of health engagement.

**General health** was measured with the item “How is your health in general?” on a 5-point scale (1 = very good, 5 = very bad) taken from the Portuguese version of the European Social Survey (Round 9) [56], with higher values indicating lower levels of health. In addition, we asked participants if they had provided home healthcare assistance, and if they already needed healthcare assistance for themselves (See Table 1).

**Fig. 1:**
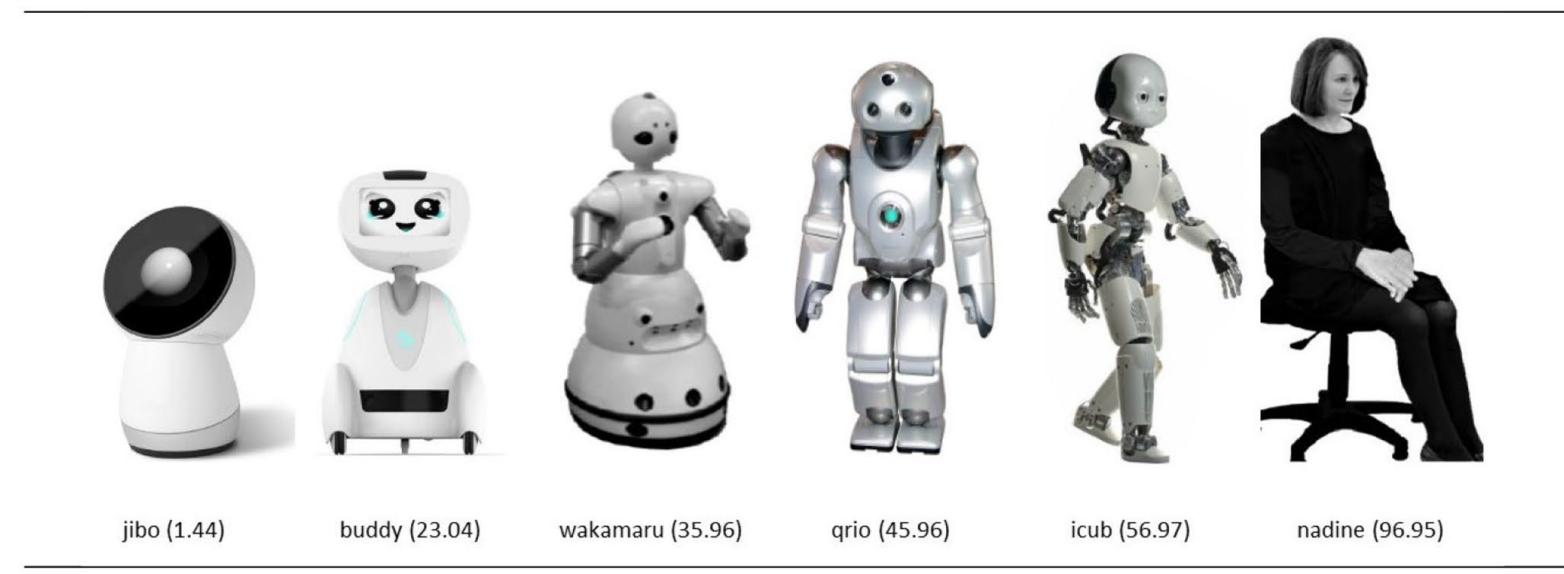
Response Format for the Selection of the Robotic Healthcare Agent Imagined and considered Suitable for the Nursing Task *Note*. The images were selected from the “Anthropomorphic Robot Database” [40]. In parentheses, the name and scores of human-likenesses.

In the conditions of a robotic healthcare agent, participants were additionally asked to select the robotic model that seemed closest to what they imagined while reading the vignette. In addition, in all conditions they were asked to select the robot most suitable for the nursing task. For these evaluations, six pictures of robots were presented, taken from “The Anthropomorphic Robot Database” [46]. These models were displayed in ascending order regarding the Human-Likeness score. Only one of these six robots could be chosen for each question (see Fig. 1).

**Sociodemographic information** included age, sex, marital status, main occupation, nationality, country of residence, and education (middle school, high school, bachelor’s degree, postgraduate degree, masters’ degree, doctoral degree).

To screen for attentive responding, participants were asked to choose one among three short descriptions of the vignette, with only one being correct. We also asked whether participants understood the vignette with the item “I understood well the situation involving the patient and Lena”, and whether the scenario was perceived as being realistic, with the item “I found the description of the situation involving the patient and Lena quite realistic”. These two items were answered on a 7-point scale (1 = strongly disagree, 7 = strongly agree).

### Procedure

The study received approval from the University’s Ethics Committee before starting to collect the data. Data was collected online through the Qualtrics platform. Participants could choose to respond either to the European or the Brazilian Portuguese versions of the survey. After agreeing with the informed consent, participants were randomly assigned by the platform to one of the eight vignettes, guaranteeing allocation concealment. The questions regarding our dependent variables were presented immediately after the participant’s exposure to the vignette, in the following order: moral acceptance and attribution of responsibility, perceived agent’s attributes (warmth, competence, trustworthiness), imagined robot and suitable robot, followed by the instruments about the variables that we aimed to control for (i.e., the potential covariates, such as NAR, moral concerns, and health engagement). For each of these measures the presentation order of the items was randomized for all participants, except for the question addressing the imagined robot, which was only shown to participants who received the vignettes including the robot as the healthcare agent. The sociodemographic information and perceived general health were requested at the end, followed by a debriefing explaining the aims of the research in more detail. Data collection occurred between 01/25/2021 and 03/06/2021. Participants took approximately 15 min to complete the survey.

### Data Analysis

The data was analyzed using the IBM SPSS Statistics software (v. 26) and is available online at the Open Science Framework project page (https://doi.org/10.17605/OSF.IO/2F9QJ).

To test our hypotheses, 3-way Analyses of Covariance (ANCOVAs) with a factorial design 2 (healthcare agent) X 2 (health framing) X 2 (ethical decision) were conducted for each of the dependent variables. In each model, we only included as covariates the sociodemographic and individual variables showing statistically significant correlations with the dependent variable (i.e., *p* < .05; see Table 2). Thus, we first tested whether the potential covariates were statistically correlated with the outcome variables. To measure the strength of these associations we computed point-biserial correlation coefficients (*r*_*pb*_) for dichotomous variables (sex, nationality), Spearman rank-order correlation coefficients (*r*_*s*_) for ordinal variables using single items (education level, general health), and Pearson’s product-moment correlation (*r*) for the composite measures (negative attitudes towards robots; harm and fairness concerns, and health engagement).

One important assumption is the independence between our independent variables and the covariates. Thus, as a further check for this selection, we also analyzed whether the eight group conditions of the independent variable were equivalent in respect to these potential covariates, although we were not expecting differences because of the participant’s random assignment to conditions. For these analyses we used Pearson’s chi-squared test (χ^2^) for categorial variables (sex, nationality), Kruskal-Wallis Test (*H*) for education level and general health, and analyses of variance (ANOVAs) for the composite measures. After selecting the potential covariates for each model, we additionally examined the assumption of homogeneity of regression slopes for the ANCOVAs, to make sure that the slope of the relationship between the covariates and the outcome variable are similar in all conditions. Finally, we ran 3-way ANOVAs to understand whether the statistically significant results of the independent variables on our outcome measures remained without including the covariates.

**Table 2:** Psychometric Properties of the Measures

	*N*	Range	*Min*	*Max*	*M*	*DP*	Cronbach’s α
Moral acceptance	524	6	2	7	4.83	1.10	0.90
Moral responsibility	524	6	1	7	3.87	1.68	0.81
Warmth	524	6	1	7	4.09	1.52	0.89
Competence	524	6	1	7	4.93	1.24	0.90
Trustworthiness	524	6	1	7	5.24	1.19	0.78
Negative Attitudes toward Robots	523	6	1	7	3.79	1.11	0.88
Harm concerns	518	5	1	6	5.04	0.80	0.79
Fairness concerns	517	5	1	6	5.15	0.76	0.78
Health engagement	502	3	2	5	3.83	0.48	0.83

## Results

### Descriptive Analysis

Most participants indicated that they understood the situation described in the vignette (93.5%) and considered the vignette realistic (84.1%). A summary of the descriptive statistics is presented in Table 3. The eight group conditions were similar in terms of their distribution by gender, χ^2^(7, *N* = 500) = 5.62, *p* = .585, and nationality, χ^2^(7, *N* = 495) = 3.33, *p* = .853, age, *F* (7, 516) = 1.64, *p* = .121, education level, *H*(7) = 5.38, *p* = .613, perceived general health, *H*(7) = 5.76, *p* = .567, and also on negative attitudes towards robots, harm and fairness concerns, and health engagement, all *F <* 1.21, *p*s > 0.23. Correlational analyses between these potential covariates and the dependent variables are displayed in Table 2.

**Table 3:** Correlations Between the Individual/Sociodemographic and the Dependent Variables

	Sex^a^	Nat^a^	Age^b^	Edu^c^	Health^c^	NAR^b^	Harm^b^	Fair^b^	He^b^
Ma	0.01	− 0.03	0.05	− 0.10*	0.003	− 0.16**	0.04	0.05	0.11*
Mr	− 0.03	− 0.11*	− 0.08	− 0.11*	0.07	0.02	− 0.06	− 0.02	− 0.05
Wa	− 0.07	− 0.07	− 0.03	− 0.06	0.01	− 0.06	0.10*	0.06	0.11*
Co	0.01	− 0.15**	− 0.11*	− 0.12**	− 0.04	− 0.17**	0.02	0.04	0.14**
Tr	0.04	− 0.03	− 0.06	− 0.06	− 0.03	− 0.20**	0.04	0.10*	0.12**

*Note*. *p < .05, **p < .01 (*two-tailed*). Ma = Moral acceptance; Mr = Moral responsibility; Wa = Warmth; Co = Competence; Tr = Trustworthiness; Sex (1 = Female; 2 = Male); Nat = Nationality (1 = Portuguese; 2 = Brazilian); Edu = Educational level; Health = general health; NAR = Negative Attitudes towards Robots; Harm = harm concern; Fair = fairness concern; He = Health engagement. ^a^Point-biserial correlation coefficient (*r*_*pb*_); ^b^Pearson’s product-moment correlation (*r*); ^c^Spearman rank-order correlation coefficient (*r*_*s*_)

Overall, the strength of the correlations was low, not exceeding 0.20. For the demographics, we found that nationality was related to competence, *r*_*pb*_ (495) = − 0.15, *p* < .001, and attribution of moral responsibility, *r*_*pb*_ (495) = − 0.11, *p* = .013. Based on their evaluation, we found that Portuguese participants judged the healthcare agent as more competent (*M* = 5.19, *SD* = 1.01) than the Brazilians (*M* = 4.81, *SD* = 1.33), and attributed more moral responsibility to the agent (*M* = 4.12, *SD* = 1.69 vs. *M* = 3.73, *SD* = 1.70). There were no gender differences on the outcomes, and age was only negatively related with perceived competence, *r* (524) = − 0.11, *p* = .014. Education level was also found to be negatively associated with evaluations of competence, *r*_*s*_ (501) = − 0.12, *p* = .006, moral acceptance, *r*_*s*_ (501) = − 0.10, *p* = .025, and attribution of responsibility to the agent, *r*_*s*_ (501) = − 0.11, *p* = .012. Regarding the individual variables, perceived general health status was not significantly related to any outcome variable. However, higher health engagement was related to perceiving the agent’s behavior as more moral acceptable, *r* (502) = 0.11, *p* = .014, and the agent’s traits as more competent, *r* (502) = 0.14, *p* = .002, warmer, *r* (502) = 0.105, *p* = .019, and trustworthy, *r* (500) = 0.12, *p* = .0095. Also, stronger harm concerns was associated with higher perceptions of warmth, *r* (518) = 0.10, *p* = .028, whereas fairness concern was related to perceiving the agent as more trustworthy, *r* (517) = 0.095, *p* = .031. Finally, participants who reported lower negative attitudes toward robots expressed higher acceptance, *r* (523) = − 0.16, *p* < .001, perceived the agent as more competent, *r* (523) = − 0.17, *p* < .001, and trustworthy, *r* (523) = − 0.20, *p* < .001.

### Hypotheses Testing

**Fig. 2:**
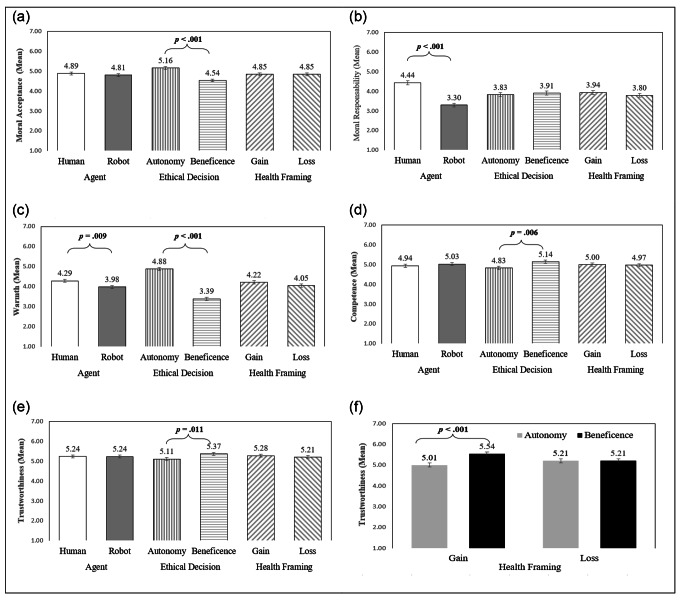
Adjusted Means for each Dependent Variable as a function of Healthcare Agent, Ethical Decision, and Health Framing *Note*. In each graph, all the values correspond to the adjusted means in the ANCOVAs as a function of the agent, ethical decision and health framing, adjusting for the following covariates included in each model: (a) education, negative attitudes towards robots, health engagement; (b) nationality, education; (c) harm concerns, health engagement; (d) nationality, age, education; (e and f) education, negative attitudes towards robots, fairness concerns, health engagement. Error bars correspond to the standard error of the means.

All the results for the main effects of the 3-way ANCOVA for each dependent variable is presented in Table 4 and the means for each condition are displayed in Fig. 2.

**Table 4:** Main Effects of the Healthcare Agent, the Health Framing, and the Ethical Decision for each Dependent Variable

		Healthcare agent			Health framing			Ethical decision		
	*df*, Error	*F*	*p*	η_p_²	*F*	*p*	η_p_²	*F*	*p*	η_p_²
M. Acceptance^Cov1^	1, 489	0.79	0.375	0.002	< 0.01	0.960	< 0.001	43.91	< 0.001	0.082
M. Responsibility^Cov2^	1, 485	63.94	< 0.001	0.116	0.86	0.353	0.002	0.35	0.554	0.001
Warmth^Cov3^	1, 492	6.78	0.009	0.014	2.25	0.135	0.005	160.12	< 0.001	0.246
Competence ^Cov4^	1, 484	0.77	0.382	0.002	0.04	0.843	< 0.001	7.66	0.006	0.016
Trustworthiness ^Cov5^	1, 487	< 0.01	0.985	< 0.001	0.44	0.509	0.001	6.46	0.011	0.013

*Note*. Covariates included in each model: Cov1 = education, negative attitudes towards robots, health engagement; Cov2 = nationality, education; Cov3 = harm concerns, health engagement; Cov4 = nationality, age, education; Cov5 = education, negative attitudes towards robots, fairness concerns, health engagement.

The 3-way ANCOVA on Moral Acceptance (controlling for the attitudes towards robots, education, and health engagement) showed a significant main effect of the ethical decision, *F*(1, 489) = 43.91, *p* < .001, ηp² = 0.082, indicating that participants in the autonomy ethical decision condition judged the agent’s behavior as more morally acceptable (*M* = 5.16, *SE* = 0.07) than participants in the beneficence/nonmaleficence condition (*M* = 4.54, *SE* = 0.07; see Fig. 2a). In contrast with the H1a, no significant main effect was found for the agent, *F*(1, 489) = 0.79, *p* = .375, ηp² = 0.002. In addition, the main effect of message framing and all the interactions among the independent variables, including the predicted ethical decision X agent type interaction, were statistically nonsignificant (*ps* > 0.05), indicating that the higher moral acceptance for an autonomy decision was similar when the agent was a human or a robot. These results were additionally confirmed when we ran a planned contrast to test H2, which showed that participants accepted the robotic agent that respected the patient’s autonomy more (*M* = 5.07, *SE* = 0.09) in comparison to the robot that prioritized beneficence/nonmaleficence (*M* = 4.54, *SE* = 0.09), *F*(1, 489) = 16.33, *p* < .001, ηp² = 0.032. These results were found above and beyond the covariates included in the model. The covariates remained statistically related to moral acceptance, after adjusting for all the other variables in the model, indicating that negative attitudes towards robots, *F*(1, 489) = 14.75, *p* < .001, ηp² = 0.029, education level, *F*(1, 489) = 8.67, *p* = .003, ηp² = 0.017, and health engagement, *F*(1, 489) = 7.48, *p* = .006, ηp² = 0.015, are significant predictors of moral acceptance judgments (*R*^2^_adj_ = 0.12). Also relevant, the statistical results of the independent variables on moral acceptance remained similar when the effects of the covariates were removed, although the explained variance decreased (*R*^2^_adj_ = 0.06).

The 3-way ANCOVA on attribution of moral responsibility, controlling for education and nationality, showed a significant main effect of the agent type, *F*(1, 485) = 63.94, *p* < .001, ηp² =0.116 (see Table 4). The evaluation of the agent’s moral responsibility was higher when the agent was a human (*M* = 4.44, *SE* = 0.10) than a robot (*M* = 3.30, *SE* = 0.10), which supports H1b (see Fig. 2b). The main effects of the ethical decision and the health-framing, as well as the interactions among the variables were all not statistically significant (*ps* > 0.05). Finally, the effects of the two covariates remained statistically significant [education, *F*(1, 485) = 5.90, *p* = .015, ηp² = 0.012, and nationality, *F*(1, 485) = 4.83, *p* = .028, ηp² = 0.010], with *R*^2^_adj_ = 0.13. Reanalysis of the results without the two covariates yielded similar findings, but slightly decreased, *R*^2^_adj_ = 0.10.

#### Healthcare Agent Attributes: Warmth, Competence, and Trustworthiness

Regarding the evaluation of the healthcare attributes, the 3-way ANCOVA on warmth, adjusting for the participant’s health engagement and harm concerns, showed significant main effects of the agent, *F*(1, 492) = 6.78, *p* = .009, ηp² = 0.014, and of the ethical decision, *F*(1, 492) = 160.12, *p* < .001, ηp² = 0.246, but no main effect of the health message framing, nor interactions between the independent variables (*ps* > 0.05). Participants perceived the human agent as being warmer (*M* = 4.28, *SE* = 0.08) than the robotic agent (*M* = 3.98, *SE* = 0.08), lending support to H1c (see Fig. 2c). Furthermore, the agent who chose to respect the patient’s autonomy was rated as being warmer (*M* = 4.88, *SE* = 0.08) than the agent who chose beneficence/nonmaleficence (*M* = 3.39, *SE* = 0.08). The effect of the covariate health engagement remained statistically significant, *F*(1, 492) = 6.69, *p* = .010, ηp² = 0.013, but the harm concerns did not reach significance. Overall, the *R*^2^_adj_ = 0.26. Conducting a 3-way ANOVA, excluding the covariates, yielded similar findings, with *R*^2^_adj_ = 0.24.

The results for competence, after controlling for age, education and nationality, revealed a significant main effect of the ethical decision, *F*(1, 484) = 7.66, *p* = .006, ηp² = 0.016, indicating that the choice for beneficence/nonmaleficence (*M* = 5.14, *SE* = 0.08) led to a higher attribution of competence to the agent than respecting the patient’s autonomy (*M* = 4.83, *SE* = 0.08; see Fig. 2d). In contrast with H1d, the main effect of the agent was statistically nonsignificant, *F*(1, 484) = 0.766, *p* = .382, ηp² = 0.002. The main effect of framing and the interactions among the independent variables were also statistically nonsignificant (*ps* > 0.05). Only the effects of the covariate nationality remained statistically significant on competence judgments, *F*(1, 484) = 4.99, *p* = .026, ηp² = 0.010. We chose to not include the negative attitudes toward robots and health engagement in the ANCOVA, because their inclusion would violate the assumption of homogeneity of the regression slope. Overall, the *R*^2^_adj_ = 0.03. Removing the covariates yields similar results, *R*^2^_adj_ = 0.01.

For trustworthiness, the 3-way ANCOVA, controlling for education, attitudes toward robots, fairness concerns and health engagement, showed a significant main effect of the ethical decision, *F*(1, 487) = 6.46, *p* = .011, ηp² = 0.013. The decision for beneficence/nonmaleficence (*M* = 5.37, *SE* = 0.07) led to a higher perception of trustworthiness than the decision to respect the patient’s autonomy (*M* = 5.11, *SE* = 0.07; see Fig. 2e). The main effects of the healthcare agent and health framing were nonsignificant (*ps* > 0.05), not supporting H1e. However, an interaction between the ethical decision and the health framing emerged, *F*(1, 487) = 6.77, *p* = .010, ηp² = 0.014. Overall, the evaluation of the agent’s trustworthiness was higher when the agent decided in favor of beneficence/nonmaleficence using a health-gain argument than in the other conditions. Simple main effects within the health-gain framing showed that perceived agent’s trustworthiness was significantly higher in the beneficence/nonmaleficence (*M* = 5.54, *SE* = 0.10) than in the autonomy condition (*M* = 5.01, *SE* = 0.11), *F*(1, 487) = 12.84, *p* < .001, ηp² = 0.026 (see Fig. 2f), although no statistical differences occurred in the loss-gain condition between the two ethical decisions, *F*(1, 487) = 0.002. The other interactions were nonsignificant (*ps* > 0.05). Finally, the effects of the covariates on trustworthiness remained significant, such as the attitudes toward robots, *F*(1, 487) = 22.46, *p* < .001, ηp² = 0.044, education, *F*(1, 487) = 9.95, *p* = .002, ηp² = 0.020, health engagement *F*(1, 487) = 7.06, *p* = .008, ηp² = 0.014, and fairness concerns, *F*(1, 487) = 6.37, *p* = .012, ηp² = 0.013. Overall, the *R*^2^_adj_ = 0.08, after adjusting for the covariates. Rerunning the analyses without adjusting for the covariates yielded similar findings, but the model with only our independent variables explained less variance, with *R*^2^_adj_ = 0.01.

### Exploratory Analyses

#### Imagined Robot and Suitable Robot

Results regarding how participants perceived the robots described in the vignette indicated that most participants selected the two robotic depictions with the highest level of human likeness score (i.e., the Icub was chosen by 26.2%, and Nadine by 24% of the participants). Because the robot appearance was ranked in terms of the degree of human likeness with only one item, we measured the relation between their choices and the dependent variables with Spearman correlations (*r*_*s*_). Overall, we found that the higher the degree of human likeness imagined, the greater was the perceived competence, *r*_*s*_ (263) = 0.23, *p* < .001, warmth, *r* (263) = 0.14, *p* = .025, trustworthiness, *r* (263) = 0.12, *p* = .045, and moral acceptability, *r* (263) = 0.14, *p* = .025.

We also asked participants to indicate which robot they considered best suited to perform healthcare related tasks. There was a clear preference for Nadine (57.3%), the robot with the higher human likeness index value, followed by Icub (18.9%), suggesting that most participants think that robots displaying high human similarity are more suitable for healthcare. No significant correlations occurred between these preferences and the dependent variables (*ps* > 0.05).

## Discussion

This study examined people’s moral judgments in the context of a healthcare setting regarding a patient who refuses medication. The healthcare agent made an ethical decision to either respect the patient’s autonomy or not accept her refusal to take the medication, given that is against the ethical principles of beneficence and nonmaleficence of the patient. Using this ethical dilemma, we examined judgments of moral acceptability and of responsibility assigned to a healthcare agent who could be a robot or a human. In addition, our study extends previous research examining how different health message framing (health-gains of taking the medication vs. health-losses of not taking the medication) impact people’s moral judgments and perceptions of the healthcare traits (warmth, competence, trustworthiness).

The results indicate that the judgments of moral acceptance about the actions of the healthcare agent are affected by the type of ethical decision, with participants judging the healthcare agent more favorable when she/it prioritizes the patient’s autonomy. Some authors claim that the principle of respect for autonomy has assumed primacy with respect to the principle of beneficence due to the rise of consumer society values [57]. Nowadays, respect for patient’s autonomy is also viewed as a core principle in healthcare ethics [26]. Interestingly, and in contrast with our first hypothesis (H1a), the actions of the human healthcare agent were judged as morally acceptable as the actions of the robot. Likewise, these results were independent of the gain or loss health arguments used to convince the patient to take the medication.

As expected, we found that moral responsibility assigned by participants to the human agent was significantly higher than the responsibility attributed to the robotic agent, thus confirming H1b. In contrast, the type of ethical decision and the framing of the message had no effect on this judgment. These results are consistent with Laakasuo et al. [9] and Kahn et al. [58] findings, in which participants also held the human agent more morally responsible than the robotic agent. The human agent must have been perceived as capable of free will, and thus more responsible for their actions than the robotic agent, given its “machine nature”. This suggests that at the moment people seem to still perceive embodied robots equipped with AI systems as having limited capabilities. However, the growing development of robots to perform complex tasks autonomously in healthcare real-world settings will pose many future challenges, including the dilemma we investigated, but also other unforeseen situations that will require appropriate decisions.

The judgments about the agent’s warmth, competence, and trustworthiness were also interesting. Warmth and competence are central elements in social perception, offering a judgment template that can also have different behavioral outcomes towards other individual or groups [1]. If people use similar concepts and explanations for the behavior of humans and robotic agents, drawing inferences of intentionality and mind, developers ought to be aware of this process in designing their systems. Our results indicated, as hypothesized (H1c), that the human agent was perceived warmer than the robotic agent. These results are in line with prior studies in which robots are judged as lacking social and emotional skills [59]. However, in contrast with our hypothesis H1d and H1e, both agents were perceived similarly in terms of competence and trustworthiness. These results are nevertheless in line with lay conceptions of competence and trust, which tend to be linked to more rational conceptions and to utilitarian roles, while warmth is an affective trait less likely to be truly felt by an artificial agent [60].

In addition, we found that the healthcare agent that respected autonomy was perceived warmer, but less competent than the agent who followed the beneficence/nonmaleficence principles by not accepting the patient’s refusal to receive medication. Thus, mind and agency attributions are not an “all or nothing” process. Although participants showed a preference for an approach that favors the autonomy of the patient, and consider the agent exhibiting that response as warmer, they also consider an agent that favors the beneficence/nonmaleficence principles to be more competent. However, both attributes are extremely important in healthcare, with competence indicating the level of benefit a healthcare agent can provide [6]. This brings us back to our initial dilemma: How should a healthcare agent respond to a patient who refuses to take medication that would benefit his/her health condition?

Several authors have proposed that when a patient refuses to adhere to a prescribed treatment or medication, the healthcare agents should disclose information about the consequences, and eventually alternative treatment options [27, 61, 62]. In our study, we introduced two different health-framing arguments to understand their role in the participants’ judgments. Although the two health arguments (gain vs. loss) showed a similar impact in most of the judgments, it was interesting to find an interaction between the ethical decision and the message framing on perceived trustworthiness. Indeed, our findings suggest that the healthcare agent following the beneficence/nonmaleficence principles was only perceived to be more trustworthy than the agent respecting the patients’ autonomy, when using arguments emphasizing the health-gain of taking the medication. Thus, participants perceived the agents who decided not to accept the patient’s will as more trustworthy if this decision is combined with arguments that were positively framed with gain-health outcomes (i.e., empathizing the health benefits for the patient). In contrast, when framing the arguments that highlight the losses of not taking the medication, the agent’s trustworthiness was similar for both ethical decisions. These results are aligned with the findings of previous studies suggesting some of the benefits of persuasive effects using gain- versus loss-framed messages, under particular circumstances [30–32, 63]. However, the only difference between these two message frames was in judgments about the trustworthiness of the healthcare agent, and like previous results the effect size was small.

All of the preceding results were found above and beyond the covariates we included in the models. Nevertheless, when testing each model with the covariates removed, the above findings remained similar. Although the variables included as covariates were not the focus of the analysis, and their association with the outcomes were relatively small, they contributed nevertheless to increase precision, and consequently to increase the explained variance on each outcome. Based on these analyses, it was interesting to find that participants with higher levels of health engagement perceived the agents as warmer and trustworthy, perhaps due to the inclusion of the agent who offered an explanation about the need for medication in all conditions, again highlighting the need to add explanations about the potential outcomes of a patient’s decision. There were also differences between countries in the judgments about the agent’s competence and on the attribution of moral responsibility. Portuguese participants perceived the agent as more competent but also attributed to her higher moral responsibility, when compared to the evaluations made by the Brazilians. To the best of our knowledge, this is the first study addressing our dilemma in these two countries. Although several studies have shown similarities between Portugal and Brazil in some cultural values [42] and moral preferences [43], with both expressing high levels of trust in nurses and doctors [44], it is possible that these findings reflect cultural differences in other dimensions that were not addressed in our design. Also interesting were the results showing that lower negative attitudes toward robots were related to perceiving the agent as more competent and trustworthy, possibly due to a wider receptivity to new technologies in general, and in Health-AI in particular. Indeed, regardless of the type of healthcare agent, all the vignettes asked participants to think about a situation in the future (year 2035), in which medical instructions were programmed through an AI system (Health-AI).

Finally, our study was also interested in evaluating the degree of human likeness attributed to the robotic agent described in the vignette. We found that more than 50% of our participants imagined a robot with the highest human likeness. In addition, the higher the selection of a robot with human likeness, the more competence, warmth, and trustworthiness was attributed to the robot. Higher human likeness was also associated with judgments of higher moral acceptability of the robot agent behaviors. Moreover, when asked to choose which robot they thought would be more suitable to perform the nursing tasks, participants showed a preference for robots with higher human likeness. These results seem to contradict the Uncanny Valley hypothesis [64], which suggests that when the appearance of a robot is almost human it may evoke feelings of unease, creepiness, or repulsion in humans. However, it is aligned with the results from a recent meta-analysis suggesting that the physical features in a robot are related to the level of anthropomorphism, which in turn seems to facilitate the interaction between humans and robots. The authors also found that higher humanlike perceptions in a robot seem to predict people’s interest in the future intention of using robots, especially when it is perceived intelligent and performs useful tasks [65]. It should also be noted that in our study participants were asked to think about a task in a context frequently depicted as a female activity [66]. Previous research also suggests that perceiving a gender identity in a robot [67] plays a key role in the judging other attributes and qualities of the robot. Moreover, this preference for a more human like robot might be aligned with the representation of a nurse than with participants’ choices for the design of a robot. However, in our perspective, we should embrace a neutral point of view and put aside gender stereotypes. These issues should also be carefully pondered during the design of the robots.

Our study also presents some limitations. Considering that we investigated a specific situation in healthcare, several of our limitations are related to generalizability, including applicability to other healthcare settings or other domains in which ethical issues and moral judgments are relevant. Indeed, moral judgments concerning the behavior and perceived attributes of the healthcare agent, and the importance of each attribute may vary depending on the situation, such as the nature of the medical procedure (e.g., need to take medication or perform a surgery) and the patient condition (e.g., chronic or acute). For example, warmth may be perceived as more relevant when dealing with chronic problems that require treatment over time (a situation that has some similarities with our dilemma), while competence seems paramount in acute ambulatory cases [5], and in our scenario the clinical condition of the patient was not mentioned. O’Keefe and Jensen [30] also pointed out that people could have different expectations regarding risk and outcome probability for different health domains, and this might translate into different message effects. Thus, it would be interesting to further explore the effects of message-framing, by addressing the role of other variables, such as the case of the patient’s medical condition of the patient, and the perceived risks associated.

Another related limitation was the restriction of the agent-patient pair to the female gender in the scenario, which may have played an important role in the participant’s judgments of both human and robot attributes and responses. Hall and Roter’s [68] meta-analysis showed gender differences in agent-patient relations, and Johanson et al. [69] brought some evidence about the gender variations of agents (humans and robots) and their influence on human-robot interaction. Thus, future studies should further investigate the role of gender in ethical dilemmas involving the patient-agent relationship in healthcare, testing if gender interferes in these judgments and how we can neutralize these gender judgments associated with professions.

A third related limitation is the use of vignettes. Some authors expressed concern for the reduced realism and difficulties in generalizing results to real world applications [70]. Although the use of vignettes is very common in research, this concern is relevant to our study. Nevertheless, we tried to minimize this concern by presenting a scenario with a situation that can be encountered in people’s everyday lives. In addition, we measured the degree of realism in the vignettes and the participant’s comprehension of the situation portrayed, to ensure that the vignettes were considered realistic and understood.

A fourth related limitation is sample representativeness. Our study used the snowball and convenience sampling methods. Although we were able to collect a large number of participants with different sociodemographic characteristics, such as age range, our sample also had a higher number of participants from certain groups, including more women than men, higher levels of education, many rating their health as generally good, with only a few stating that they had already needed healthcare assistance for themselves. Moreover, few participants were health professionals, although almost half reported having already provided healthcare assistance. Thus, the findings are limited to the characteristics of the sample and the limitation of our sampling method. It would be relevant for future studies to include more healthcare professionals and those working in ethics and robotics, including a balanced gender sample. In addition, understanding the judgments of participants with less education and from those requiring healthcare assistance will be paramount in this line of research. Finally, our study was collected among Portuguese and Brazilian participants,  in contrasts to most studies, conducted mostly in Western and often in English-speaking nations [71]. However, culture may also play a relevant role in moral judgments. Thus, it would be relevant to investigate further how people judge these ethical dilemmas in healthcare contexts in other cultures, by also considering the role of cultural dimensions.

## Conclusion

One of the arguments for the use of robots in healthcare settings is its cost-efficiency, with stakeholders advocating that a robot can perform simple, repetitive tasks, such as delivering medication to patients. However, what should a robot (or a human) do when a patient refuses medication? What would you prefer if you were the target patient? How should these socially assistive robots be programmed in case these dilemmas arise? How should they communicate with patients the importance of the prescribed medication?

Upon addressing the above questions, our results suggested that regardless of the healthcare agent type, people seem to evaluate more favorably those who respect the patients’ autonomy. Indeed, it was the decision considered more morally acceptable and the healthcare attributes were also perceived as warmer. Although our target participants were not healthcare professionals, our results are also aligned with the ICN Code of Ethics for Nurses [26]. On the other hand, the healthcare agent who favored the patient’s health status, disregarding the patient’s wishes, was perceived to be more competent and trustworthy. These results underline how a simple task of providing medication can became an ethical conundrum. People tend to prefer a warm, competent, and trustworthy nurse. However, to be warm, the nurse needs to accept decisions that may jeopardize the patient’s health. In contrast, to be perceived as more competent and trustworthy, the healthcare agent should not accept the patient’s wishes. Thus, based on these judgments, how should a robot be programmed to solve the inconsistencies? In our view, to overcome this dilemma, one might need to reframe it. It is incumbent on the healthcare agent to welcome the patient’s non-adherence, while interacting with the patient in a manner that respects the practical ethics of care and the logic of caregiving and understanding what the patient is attempting to communicate through her behavior [72]. The establishment of a mutual relationship is important for the co-construction of a procedure that, on one hand, favors the patient’s health from a biopsychosocial perspective [20], while crucially being able to balance the idiosyncrasies of the patient with the technical concerns of the healthcare agent. Investigating how communication should unfold will be very important. Thus, an important finding of our study originated from the manipulation of the gain vs. loss framing. Indeed, healthcare agents should provide the necessary information and courses of action aiming to also follow the principle of beneficence. In our results, we found that a healthcare agent that argues about the health benefits of taking the medication combined with not accepting the patient’s will is perceived as more trustworthy. This result is also in line with the findings of [62], who proposed a multidimensional perspective of the principle of autonomy in the context of a nursing home. The authors found that both nurses and physicians have different notions about this principle depending on the circumstances and highlighted that the liberal acceptance of a patient’s wishes when it compromises the principle of beneficence, might correspond to negligence, instead of good care. Under these circumstances, the caregiver must consider the patient’s limitations, try to understand their underlying motivations and to reason about their needs and wishes. Although our study was not conducted specifically with healthcare professionals, we believe it will be important for future studies to address their perspectives about these dilemmas applied to nursing robots and examine if our results also hold using arguments framed as health-gains instead of health-loss.

Also noteworthy is that the participants considered the human healthcare agent to be more morally responsible than the robotic agent, regardless of the ethical decision or the health framing. The fact that robots are less harshly judged for its actions than the human healthcare reflects the current gray area related to the legal implications in determining who should be held responsible if the robot’s actions cause harm to a patient, either by action or inaction. The dilemma addressed in our study seemed to be difficult for healthcare professionals. Designing a robot to make such a decision might even be more complex. However, the possibility that a patient refuses to take medication is quite realistic. The number of humanoid service robots deployed in healthcare systems has been increasing, has accelerated during the COVID-19 pandemic [73], and we believe that it will continue to grow, with more autonomous robots being designed to make decisions. Thus, understanding how people respond to healthcare agents and to social service robots designed for these tasks in a way that will be able to preserve beneficence/nonmaleficence without disrespecting the patient’s autonomy is of the greatest importance. In the current Industrial Revolution 4.0 more efforts should be made to deploy ethical robots for patient health with care.

## References

[1] Cuddy AJC, Fiske ST, Glick P (2007). The BIAS map: behaviors from intergroup affect and stereotypes. J Personal Soc Psychol.

[2] Fiske ST, Cuddy AJC, Glick P, Xu J (2002). A model of (often mixed) stereotype content: competence and warmth respectively follow from perceived status and competition. J Personal Soc Psychol.

[3] Malle BF (2021). Moral Judgments. Ann Rev Psychol.

[4] Ullman D, Malle BF (2018) What does it mean to trust a robot?: Steps toward a multidimensional measure of trust. Companion of the 2018 ACM/IEEE International Conference on Human-Robot Interaction: ACM. 10.1145/3173386.3176991

[5] Howe LC, Leibowitz KA, Crum AJ (2019). When your doctor “gets it” and “gets you”: the critical role of competence and warmth in the patient-provider interaction. Front Psychiatry10.

[6] Melchert T (2020). Foundations of health service psychology: an evidence-based biopsychosocial approach.

[7] London AJ (2022). For the common good: philosophical foundations of research ethics.

[8] Beauchamp T, Childress J (2019). Principles of biomedical ethics.

[9] Laakasuo M, Palomäki J, Kunnari A, Rauhala S, Drosinou M, Halonen J, Lehtonen N, Koverola M, Repo M, Sundvall J, Visala A, Francis KB (2023) Moral psychology of nursing robots: exploring the role of robots in dilemmas of patient autonomy. Eur J Soc Psychol 53:108–128. 10.1002/ejsp.2890

[10] Russell S, Norvig P (2021). Artificial intelligence: a modern approach.

[11] Rasche C, Pfannstiel M, Da-Cruz P, Mehlich H (2016). Digitaler gesundheitswettbewerb: Strategien, geschäftsmodelle, kompetenzanforderungen&nbsp;[Digital health competition: Strategies, business models, competence requirements]. Digitale Transformation von Dienstleistungen im Gesundheitswesen I&nbsp;[Digital Transformation of Services in Healthcare I].

[12] Fong T, Nourbakhsh I, Dautenhahn K (2003). A survey of socially interactive robots. Robot Auton Syst.

[13] Devillers L (2021) Human–robot interactions and affective computing: the ethical implications. In: von Braun J, Archer MS, Reichberg G, M., SS (eds) Robotics, AI, and humanity: Science, ethics, and policy. Springer International Publishing, pp 205–211

[14] Pepito JA, Locsin R (2018). Can nurses remain relevant in a technologically advanced future?. Int J Nurs Sci.

[15] Locsin RC, Ito H (2018). Can humanoid nurse robots replace human nurses?. J Nurs.

[16] Riether N, Hegel F, Wrede B, Horstmann G (2012) Social facilitation with social robots?. Proceedings of the 7th Annual ACM/IEEE International conference on Human-Robot Interaction - HRI ’12, pp 41–47. 10.1145/2157689.2157697

[17] Esterwood C, Robert LP (2021). A systematic review of human and robot personality in health care human-robot interaction. Front Rob AI.

[18] Paiva A, Correia F, Oliveira R, Santos F, Arriaga P, Lugrin B, Pelachaud C, Taum D (2021). Empathy and prosociality in social agents. The handbook on socially interactive agents.

[19] Kahn PH, Reichert AL, Gary HE, Kanda T, Ishiguro H, Shen S et al (2011) The new ontological category hypothesis in human-robot interaction. Proceedings of the 6th International Conference on Human-Robot Interaction - HRI’11, pp 159–160. 10.1145/1957656.1957710

[20] Hall J, Roter D, Friedman H (2011). Physician-patient communication. The Oxford Handbook of Health psychology.

[21] Benyamini Y, Friedman H (2011). Health and illness perceptions. The Oxford Handbook of Health psychology.

[22] Kaplan RM, Friedman H (2011). Uncertainty, variability, and resource allocation in the health care decision process. The Oxford Handbook of Health psychology.

[23] Nunes L, Amaral M, Gonçalves R (2005) Código deontológico do enfermeiro: dos comentários à análise de casos [Deontological Code of Nursing: From comments to case analysis]. Lisboa: Ordem dos Enfermeiros. https://www.ordemenfermeiros.pt/media/8889/codigodeontologicoenfermeiro_edicao2005.pdf Accessed 30 October 2022

[24] Conselho Federal de Enfermagem (1999) Resolução COFEN-218/1999 [COFEN Resolution – 218/1999]. http://www.cofen.gov.br/resoluo-cofen-2181999_4264.html. Accessed 30 October 2022

[25] ten Have H, Neves MCP (2021). Nursing Ethics. Dictionary of Global Bioethics.

[26] International Council of Nurses I. The ICN Code of Ethics for Nurses: Revised 2021. International Council of Nurses. https://www.icn.ch/system/files/2021-10/ICN_Code-of-Ethics_EN_Web_0.pdf

[27] Nunes V, Neilson J, O’Flynn N, Calvert N, Kuntze S, Smithson H (2009). Medicines adherence: involving patients in decisions about prescribed medicines and supporting adherence.

[28] Rothman AJ, Salovey P (1997). Shaping perceptions to motivate healthy behavior: the role of message framing. Psych Bull.

[29] Tversky A, Kahneman D (1981). The framing of decisions and the psychology of choice. Science.

[30] O’Keefe DJ, Jensen JD (2007). The relative persuasiveness of gain-framed loss-framed messages for encouraging disease prevention behaviors: a meta-analytic review. J Health Communication.

[31] Nabi RL, Walter N, Oshidary N, Endacott CG, Love-Nichols J, Lew ZJ (2019). Can emotions capture the elusive gain-loss framing effect? A meta-analysis. Communication Res.

[32] Gallagher KM, Updegraff JA (2011). Health message framing effects on attitudes, intentions, and behavior: a meta-analytic review. Ann Behav Med.

[33] Dai Z, MacDorman K (2018). The doctor’s digital double: how warmth, competence, and animation promote adherence intention. PeerJ Comput Sci.

[34] Malle BF, Scheutz M, Arnold T, Voiklis J, Cusimano C (2015) Sacrifice one for the good of many? Proceedings of the 10th Annual ACM/IEEE International Conference on Human-Robot Interaction: ACM/IEEE, pp 117–124. 10.1145/2696454.2696458

[35] Malle BF, Scheutz M, Forlizzi J, Voiklis J (2016) Which robot am I thinking about? The impact of action and appearance on people’s evaluations of a moral robot. Proceedings of the 11th ACM/IEEE International Conference on Human-Robot Interaction (HRI). ACM/IEEE. p. 125–132. 10.1109/HRI.2016.7451743

[36] Anderson M, Anderson SL, Armen C (2006) MedEthEx: A prototype medical ethics advisor. Proceedings of the 18th Conference on Innovative Applications of Artificial Intelligence, Vol. 2. AAAI Press, Boston, pp 1759–1765. 10.5555/1597122.1597134

[37] Anderson M, Anderson SL (2018) GenEth: A general ethical dilemma analyzer. Paladyn, Journal of Behavioral Robotics 9(1): 337 – 57. 10.1515/pjbr-2018-0024

[38] Sasson S (2000). Beneficence versus respect for autonomy. J Gerontol Soc Work.

[39] Spatola N, Wudarczyk OA (2020). Implicit attitudes towards robots predict explicit attitudes, semantic distance between robots and humans, anthropomorphism, and prosocial behavior: from attitudes to human-robot interaction. Int J Social Robot.

[40] Tilburt JC, James KM, Jenkins SM, Antiel RM, Curlin FA, Rasinski KA (2013). "Righteous minds” in health care: measurement and explanatory value of social intuitionism in accounting for the moral judgments in a sample of U.S. physicians. PLoS ONE.

[41] Graffigna G, Barello S (2018). Spotlight on the patient health engagement model (PHE model): a psychosocial theory to understand people’s meaningful engagement in their own health care. Patient Prefer Adherence.

[42] Inglehart R, Welzel C (2005). Modernization, cultural change, and democracy: the human development sequence.

[43] Awad E, Dsouza S, Shariff A, Rahwan I, Bonnefon J-F (2020) Universals and variations in moral decisions made in 42 countries by 70,000 participants. Proceedings of the National Academy of Sciences 117(5): 2332–2337. 10.1073/pnas.1911517117 10.1073/pnas.1911517117 PMC7007553 31964849

[44] Wellcome Global Monitor (2018) Appendix C: Country-level data. https://wellcome.org/reports/wellcome-global-monitor/2018/appendix-country-level-data. Accessed January 2021

[45] Hester N, Xie SY, Hehman E (2021). Little between-region and between-country variance when people form impressions of others. Psychol Sci.

[46] Phillips E, Zhao X, Ullman D, Malle BF (2018) What is human-like? Decomposing robots’ human-like appearance using the Anthropomorphic roBOT (ABOT) Database. Proceedings of the 2018 ACM/IEEE International Conference on Human-Robot Interaction, pp. 105–113. 10.1145/3171221.3171268

[47] Broadbent E, Kumar V, Li X, Sollers J 3rd, Stafford RQ, MacDonald BA et al (2013) Robots with display screens: a robot with a more humanlike face display is perceived to have more mind and a better personality. PLoS ONE 8(8):e72589. 10.1371/journal.pone.0072589 10.1371/journal.pone.0072589 PMC3755978 24015263

[48] Christoforakos L, Gallucci A, Surmava-Große T, Ullrich D, Diefenbach S (2021). Can robots earn our trust the same way humans do? A systematic exploration of competence, warmth, and anthropomorphism as determinants of trust development in HRI. Front Rob AI.

[49] Faul F, Erdfelder E, Lang A-G, Buchner A (2007). G*Power 3: a flexible statistical power analysis program for the social, behavioral, and biomedical sciences. Behav Res Methods.

[50] Piçarra N, Giger JC (2018). Predicting intention to work with social robots at anticipation stage: assessing the role of behavioral desire and anticipated emotions. Comput Hum Behav.

[51] Oliveira R, Arriaga P, Correia F, Paiva A (2019) The stereotype content model applied to human-robot interactions in groups. 14th ACM/IEEE International Conference on Human-Robot Interaction (HRI) pp. 123 – 32. 10.1109/HRI.2019.8673171

[52] Diamantopoulos A, Sarstedt M, Fuchs C, Wilczynski P, Kaiser S (2012). Guidelines for choosing between multi-item and single-item scales for construct measurement: a predictive validity perspective. J Acad Mark Sci.

[53] Piçarra N, Giger JC, Pochwatko G, Gonçalves G (2015). Validation of the portuguese version of the negative Attitudes towards Robots Scale. Eur Rev Appl Psychol.

[54] Graham J, Nosek BA, Haidt J, Iyer R, Koleva S, Ditto PH (2011). Mapping the moral domain. J Personal Soc Psychol.

[55] Duke CC, Lynch WD, Smith B, Winstanley J (2015). Validity of a new patient engagement measure: the Altarum Consumer Engagement (ACE) measure. The Patient.

[56] European Social Survey (2018) ESS Round 9 Source Questionnaire. ESS ERIC Headquarters, University of London. https://www.europeansocialsurvey.org/data/download.html?r=9

[57] Ashcroft RE (2012) Health technology assessment. Encyclopedia of Applied Ethics, 2nd edn. Elsevier, pp 556–565

[58] Kahn PH, Severson RL, Kanda T, Ishiguro H, Gill BT, Ruckert JH et al (2012) Do people hold a humanoid robot morally accountable for the harm it causes? Proceedings of the seventh annual ACM/IEEE international conference on Human-Robot Interaction, ACM Press, pp 33–40. 10.1145/2157689.2157696

[59] de Graaf MMA, Ben Allouch S (2016) Anticipating our future robot society: The evaluation of future robot applications from a user’s perspective. 25th IEEE International Symposium on Robot and Human Interactive Communication (RO-MAN), IEEE, pp 755–762, 10.1109/ROMAN.2016.7745204

[60] Loughnan S, Haslam N (2007). Animals and androids: implicit associations between social categories and nonhumans. Psychol Sci.

[61] Varkey B (2021). Principles of clinical ethics and their application to practice. Med Principles Pract.

[62] van Thiel GJ, van Delden JJ (2002). The principle of respect for autonomy in the care of nursing home residents. Nurs Ethics.

[63] Dorison CA, Lerner JS, Heller BH, Rothman AJ, Kawachi II, Wang K (2022). In COVID-19 Health Messaging, loss framing increases anxiety with little-to-No concomitant benefits: experimental evidence from 84 countries. Affect Sci.

[64] Mori M (1970). The uncanny valley. Energy.

[65] Blut M, Wang C, Wünderlich NV, Brock C (2021). Understanding anthropomorphism in service provision: a meta-analysis of physical robots, chatbots, and other AI. J Acad Mark Sci.

[66] Polit DF, Beck CT (2008) Is there gender bias in nursing research? Res Nurs Health. 2008;31(5):417 – 27. 10.1002/nur.20276 10.1002/nur.20276 18324681

[67] Eyssel F, Hegel F (2012). (S)he’s got the look: gender stereotyping of robots. J Appl Soc Psychol.

[68] Hall JA, Roter DL (2002) Do patients talk differently to male and female physicians? Patient Education and Counseling 48(3): 217 – 24. 10.1016/s0738-3991(02)00174-x 10.1016/s0738-3991(02)00174-x 12477606

[69] Johanson DL, Ahn HS, Broadbent E (2020). Improving interactions with healthcare robots: a review of communication behaviours in social and healthcare contexts. Int J Social Robot.

[70] Aguinis H, Bradley KJ (2014). Best practice recommendations for designing and implementing experimental vignette methodology studies. Organizational Res Methods.

[71] Newson M, Buhrmester M, Xygalatas D, Whitehouse H, Go WILD, Not WEIRD (2021). Journal for the Cognitive Science of Religion.

[72] Pettersen T (2011). The ethics of care: normative structures and empirical implications. Health Care Anal.

[73] Ozturkcan S, Merdin-Uygur E (2021). Humanoid service robots: the future of healthcare?. J Inform Technol Teach Cases.

